# Computational Identification of MoRFs in Protein Sequences Using Hierarchical Application of Bayes Rule

**DOI:** 10.1371/journal.pone.0141603

**Published:** 2015-10-30

**Authors:** Nawar Malhis, Eric T. C. Wong, Roy Nassar, Jörg Gsponer

**Affiliations:** 1 Centre for High-Throughput Biology, University of British Columbia, Vancouver, BC, Canada; 2 Department of Biochemistry and Molecular Biology, University of British Columbia, Vancouver, BC, Canada; University of Alberta, CANADA

## Abstract

**Motivation:**

Intrinsically disordered regions of proteins play an essential role in the regulation of various biological processes. Key to their regulatory function is often the binding to globular protein domains via sequence elements known as molecular recognition features (MoRFs). Development of computational tools for the identification of candidate MoRF locations in amino acid sequences is an important task and an area of growing interest. Given the relative sparseness of MoRFs in protein sequences, the accuracy of the available MoRF predictors is often inadequate for practical usage, which leaves a significant need and room for improvement. In this work, we introduce MoRF_CHiBi_Web_, which predicts MoRF locations in protein sequences with higher accuracy compared to current MoRF predictors.

**Methods:**

Three distinct and largely independent property scores are computed with component predictors and then combined to generate the final MoRF propensity scores. The first score reflects the likelihood of sequence windows to harbour MoRFs and is based on amino acid composition and sequence similarity information. It is generated by MoRF_CHiBi_ using small windows of up to 40 residues in size. The second score identifies long stretches of protein disorder and is generated by ESpritz with the DisProt option. Lastly, the third score reflects residue conservation and is assembled from PSSM files generated by PSI-BLAST. These propensity scores are processed and then hierarchically combined using Bayes rule to generate the final MoRF_CHiBi_Web_ predictions.

**Results:**

MoRF_CHiBi_Web_ was tested on three datasets. Results show that MoRF_CHiBi_Web_ outperforms previously developed predictors by generating less than half the false positive rate for the same true positive rate at practical threshold values. This level of accuracy paired with its relatively high processing speed makes MoRF_CHiBi_Web_ a practical tool for MoRF prediction.

**Availability:**

http://morf.chibi.ubc.ca:8080/morf/.

## Introduction

Intrinsically disordered regions (IDRs) are protein segments that do not adopt a unique 3D structure under physiological conditions [1–3]. Of particular interest to many researchers are relatively short segments within IDRs that can undergo disorder-to-order transitions during binding, which are known as molecular recognition features (MoRFs), a term that had only been coined a decade ago but had quickly gained recognition [4–8]. Interactions mediated by MoRFs are important and play key roles in regulatory processes and in signal transduction [3], as their structural flexibility grants MoRFs the ability to mold into a precise fit for a given binding surface and, thereby, achieve high interaction specificity, which is often desirable for protein interactions in signaling pathways [9]. Furthermore, long MoRFs form large interaction surfaces with their partners, which contain many specificity-enhancing electrostatic interactions [10]. Alternative splicing is another mechanism of regulating protein interactions that involves MoRFs. Exons that are alternatively spliced in a tissue specific manner are enriched with MoRFs [11], which allow protein interaction networks to be rewired by altering the availability of MoRF motifs in protein isoforms, and thus modulate the signal–integrating pathways.

Given the properties and functional importance of MoRFs, their identification has become an important computational challenge and several computational methods have been developed in recent years for that purpose, including the programs ANCHOR [12], MoRFpred [13], MFSPSSMpred [14], and DISOPRED3 [15]. ANCHOR and MoRFpred are among the most used MoRF predictors. ANCHOR, a downloadable and fast predictor, makes predictions based on the estimation of interaction energies between the residues in the protein sequence. ANCHOR searches for sequences in IDRs that have low stabilization energy on their own but have the propensity to interact with globular proteins. On the other hand, the web-based predictor MoRFpred predicts MoRFs using a linear kernel SVM-based approach to combine various properties extracted from the protein sequence, including physicochemical properties of amino acids, conservation of residues, predicted solvent accessibility of residues, and scores from five IDR predictors. MFSPSSMpred is a web-based predictor employing a SVM model with a radial basis function (RBF) kernel that utilizes sequence properties and conservation. The currently available prediction engine for MFSPSSMpred was trained on almost all known MoRF sequences, which limits the data available for external evaluation. However, an unreleased version of MFSPSSMpred that was trained and evaluated using the same training and test datasets used by MoRFPred showed a performance that was approximately equal to that of MoRFpred in terms of AUC, with a slight underperformance at low false positive rates (FPRs) [14]. DISOPRED3, just like MFSPSSMpred, is a web-based SVM-RBF model that was trained on all known MoRF sequences. In addition to sequence property and conservation, DISOPRED3 also relies on disorder predictions generated independently by a neural network model.

We recently introduced MoRF_CHiBi_, a downloadable predictor for identifying MoRF sequences of length between 5 and 25 residues using windows with up to 40 residues in size [16]. Conveniently, MoRF_CHiBi_’s exclusive reliance on local physicochemical properties of amino acids and the lack of dependence on any external component predictors makes it a good candidate for incorporating into a more complex predictor for high precision MoRF identification.

In this work, we introduce MoRF_CHiBi_Web_, the most accurate computational method to date for the prediction of MoRFs in protein sequences. MoRF_CHiBi_Web_ utilizes Bayes rule to combine the output of MoRF_CHiBi_ with the output of an intermediate predictor, MoRF_DC_, constructed from protein disorder and conservation information. The integration of disorder and conservation information resulted in significant improvements in the prediction quality of MoRF_CHiBi_Web_ that is reflected in its ability to predict short MoRFs of up to 30 residues in length with an AUC of 0.87.

## Methods

**Fig 1:**
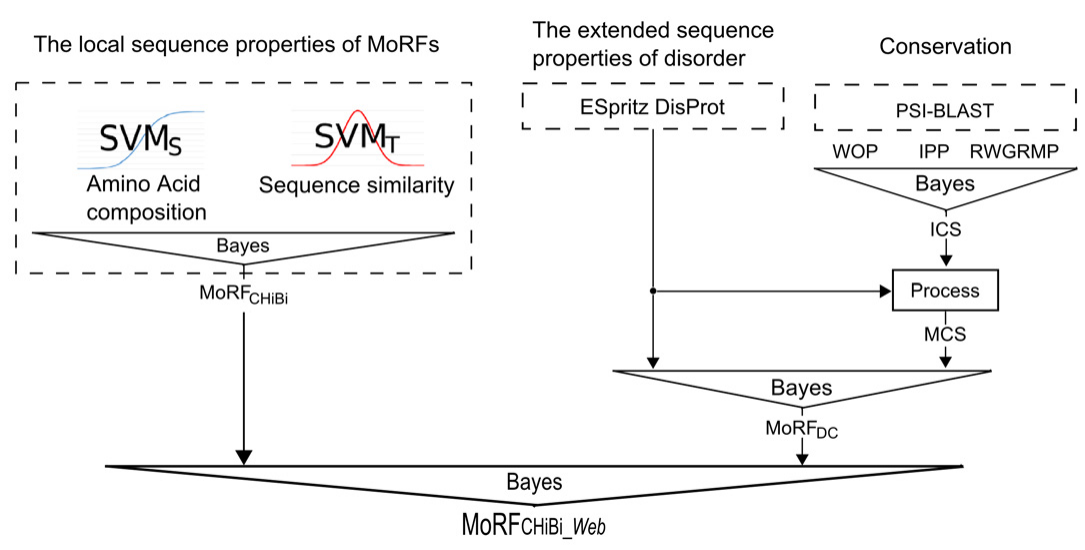
MoRF_CHiBi_Web_ structure. The three components used by MoRF_CHiBi_Web_ are MoRF_CHiBi_ predictions, predictions of disorder by ESpritz with the DisProt option, and conservation predictions using PSI-BLAST. These three components are combined by using Bayes rules at multiple levels.

Three distinct and largely independent sources of information are used in MoRF_CHiBi_Web_ (Fig 1).

*A) Sequence similarity and contrast in amino acid composition*: In addition to sequence similarities between MoRFs, one can also exploit the fact that the amino acid composition of MoRFs is different from that of the general protein population [12, 13, 16] and contrasts most with the MoRFs’ flanking sequences (Flanks). MoRF_CHiBi_ predicts MoRFs using two SVM models with two different noise tolerant kernels, SVM_S_ and SVM_T_. SVM_S_, with a sigmoid kernel, extracts information related to the general contrast in amino acid composition between MoRFs and their Flanks. On the other hand, SVM_T_ relies on a RBF kernel to identify sequence similarities between regions in a query sequence and MoRFs of the training set. Thus, in this work, MoRF_CHiBi_ is used to generate a MoRF propensity score based on these two features.

*B) Disorder*: The residue composition of MoRFs is more similar to structured domains than to their surrounding disordered regions [7, 12, 13, 16]. As MoRF_CHiBi_ relies solely on local sequence information, some of its identified MoRFs do not lie within disordered protein regions. Therefore, MoRF_CHiBi_Web_ utilizes information on the level of protein disorder over longer regions encompassing putative MoRFs to improve its prediction accuracy. However, identifying such long stretches of disorder sequences requires disorder predictors that are designed for that purpose. Most disorder predictors are designed to identify short disorder segments and therefore generate low propensity scores at MoRF sites; in other words, their prediction scores dip in the vicinity of MoRFs [17]. Disorder predictors that target long disorder segments generate disorder propensity scores that do not dip at MoRF locations, so they tend to identify the entirety of a MoRF-harbouring IDR as disordered (more on this in Methods section: Selecting an appropriate disorder predictor).

*C) Conservation*: As functional elements, MoRF residues are more conserved on average than IDR residues [18, 19]. Furthermore, we assumed that the conservation is more pronounced for the subset of MoRF residues that are directly involved in binding and are part of the interaction interfaces (we documented the validation of this assumption in the Methods section “Binding residues and conservation“). Under this assumption, a MoRF consists of some highly conserved residues that are interspersed among less conserved residues. However, MoRFs would be computationally easier to identify if the high conservation scores extend throughout the entire MoRF sequence. Thus, we generated a *MoRF conservation propensity score*, *mcs*, such that the higher conservation scores of putative MoRF residues are extended to their neighboring residues (more on this in Methods section “Conservation propensity”).

These three sources of information–sequence similarity/contrast in amino acid composition, disorder, and conservation–are used in two steps to construct MoRF_CHiBi_Web_. First, an intermediate predictor, MoRF_DC_, utilizes Bayes rule to integrate disorder and conservation information into MoRF propensity scores. Then, Bayes rule is used again to combine the propensity scores of MoRF_DC_ and MoRF_CHiBi_ into the final MoRF_CHiBi_Web_ scores.

In general, accumulating probabilities with Bayes rule should not be preceded by data processing. However, when Bayes rule is used to combine different propensities that do not reflect real probabilities, unjustified extreme values close to zero or one tend to dominate the prediction outcome. To reduce this effect, we transformed each set of input propensity scores from its unknown distribution into a Gaussian distribution with no extreme values (more on this in the Methods section: “Transforming data to normality”).

### Datasets

Disfani et al. [13] collected a large set of structures containing protein-peptide interactions from the Protein Data Bank (PDB) deposited during or before March 2008 and filtered them on a number of principles to identify a set of 840 protein sequences. Each of these sequences includes a single peptide region with 5 to 25 residues presumed to be a MoRF. They divided these 840 sequences into a training set (TRAINING) with 421 sequences and the first test set with 419 sequences. Using similar criteria, Disfani et al. also collected a second test set from more recent PDB entries deposited between January 1st and March 11th, 2012. It consists of 45 sequences. All test sequences have less than 30% identity to those in TRAINING. We decided to use the same training set (TRAINING) used by Disfani et al. and Malhis and Gsponer [16] so that the performance of our predictor could be directly compared to theirs. TRAINING has a total of 245,984 residues including 5,396 MoRF residues. We evaluated our predictor using three test sets. The first test set, TEST464, includes all of the 464 sequences of the first and second test sets collected by Disfani et al. with a total of 296,362 residues including 5,779 MoRF residues. Although TEST464 is composed of sequence sets that have previously been used to test the performance of MoRF predictors [13, 14, 16], it may not be ideal for the task for two main reasons; Firstly, TEST464 is not free of redundancy as more than a third of its sequences share 90% or more identity with at least one other sequence in the set. Secondly, the selection procedure used by Disfani et al. does not verify that the identified peptides are disordered when alone in solution, i.e. that they are *bona fide* MoRFs. Therefore, we assembled an additional test set (EXP53) that includes only sequences that have been experimentally validated for their disordered properties in isolation. This second test set contains 53 non-redundant sequences that are sourced from four datasets consisting of MoRFs that have been experimentally validated to be disordered in isolation: First, a set of 8 sequences collected by Disfani et al. [13]. Second, a set of 40 sequences with short MoRFs of 30 residues or less which was used to train ANCHOR [12]. Third, a set of 26 sequences with long MoRFs of more than 30 residues that was also collected by Mészáros et al. [12]. And finally, a set of 21 sequences that was collected by Jones et al. [15]. We combined these four sets, and then removed sequences with more than 30% identity to those in TRAINING and redundant sequences with more than 30% identity with each other. We removed one additional sequence (Q51918|PDB:1YMH_E) because we could not find evidence for a disordered state in separation, its PDB structure resembles a globular domain, and the chain in the PDB structure, 1YMH_E, matched poorly to the protein sequence. Overall, EXP53 has 25,186 residues including 2,432 MoRF residues, which can be divided into 729 from sections with up to 30 residues identified as MoRF residues based one or more PDB structures and 1,703 from sections longer than 30 residues (please see “S2 Text”). The third test set, EXP9, consists of 9 sequences with experimentally validated MoRFs that were collected by Jones et al. These sequences are not homologous to any sequences used in the training of MFSPSSMpred, MoRFpred and ANCHOR. Hence, these 9 sequences do not overlap with our training data either, since we used the same training set as MoRFpred. EXP9 contains a total of 2,209 residues, including 12 MoRF regions with 163 MoRF residues. This third test set is used to compare prediction quality with MFSPSSMpred and DISOPRED3.

### Selecting an appropriate disorder predictor

**Fig 2:**
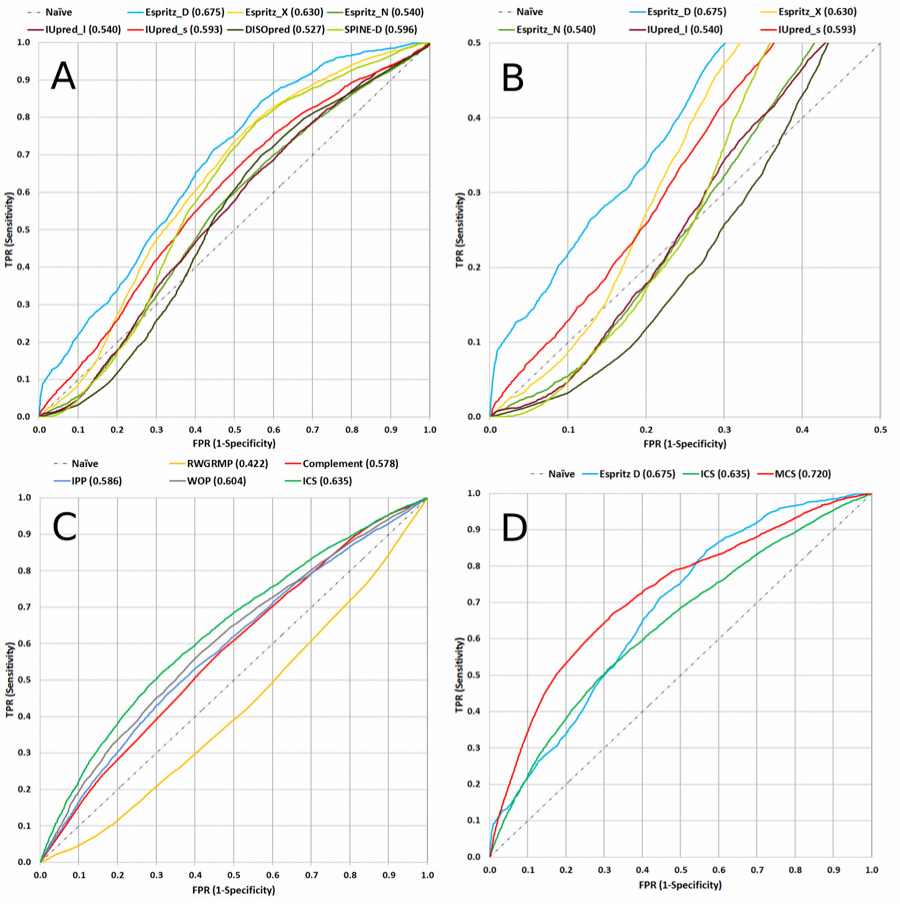
ROC_MoRF_ curves of multiple prediction tools. Vertical axis is the true positive rate (TPR) and horizontal axis is the false positive rate (FPR). AUC_MoRF_ values are in parentheses next to each label. (A) ROC_MoRF_ curves of seven IDP predictors: DISOPRED (in black), ESpritz with the DisProt option (ESpritz_D in blue), ESpritz with the X-Ray option (ESpritz_X in yellow), ESpritz with the NMR option (ESpritz_N in green), IUPred with the long option (IUPred_l in brown), IUPred with the short option (IUPred_s in red), and SPINE-D (in light green). (B) The lower left corner of the ROC_MoRF_ curves for the seven IDP predictors. (C) ROC_MoRF_ curves of IPP (in blue), WOP (in gray), RWGRMP (in yellow) and its complement (Complement, in red). The Initial conservation propensity score, *ics*, (in green) was generated by joining IPP, Complement and WOP using Bayes rule. (D) ROC_MoRF_ curves of ESpritz_D (in blue), *ics* (in green) and *mcs* (in red). *mcs* values are generated by processing *ics*.

Several computational tools are available to predict the locations of disordered segments in proteins, including DISOPRED [20], ESpritz [21], IUPred [22, 23], and SPINE-D [24]. We evaluated the above disorder predictors on their performance in distinguishing MoRFs from non-MoRF residues by calculating the AUC_MoRF_ (area under the ROC_MoRF_ curve). As mentioned before, we require a disorder predictor that identifies MoRF residues as disordered. Our selection was made based on two measures: a high AUC_MoRF_ value and a high true positive rate (TPR) at the lower left corner of ROC_MoRF,_ which together represent high confidence predictions. Based on the ROC_MoRF_ curves and AUC_MoRF_ values generated by the above IDP predictors on TRAININIG (Fig 2A and 2B), ESpritz_D (ESpritz with the DisProt option) was selected because it provides the highest overall AUC_MoRF_ (0.675) and the highest TPR at the lower left corner of the ROC_MoRF_ curve.

### Conservation propensity scores

First, three different conservation propensity scores from the PSI-BLAST [25] position specific scoring matrix (PSSM) files were calculated and tested for their ability to identify MoRFs: (1) the information per position (IPP), (2) the relative weight of gapless real matches to pseudocounts (RWGRMP), and (3) the weighted observed percentage of the query sequence residue rounded down (WOP). Alignment databases and PSI-BLAST parameters were selected to maximize AUC_MoRF_ on TRAINING for each of the three scores independently (i.e. IPP, RWGRMP, and WOP). We tested the NCBI non-redundant, UniRef90, and UniProtKB/Swiss-Prot databases with and without filtering out coiled-coil and transmembrane regions using the program Pfilt [26]. We used three different amino acid substitution matrices (i.e. BLOSUM45, BLOSUM62, and BLOSUM80) in combination with a number of alignment iterations between 2 and 5. Note that when an AUC_MoRF_ was less than 0.5, we used the propensity score’s complement. Thus, our selection criterion was to maximize the absolute value of |AUC_MoRF_—0.5|. For IPP and WOP, we selected UniRef90, BLOSUM62, and 2 alignment iterations, which resulted in AUC_MoRF_ values of 0.586 and 0.604 respectively. For RWGRMP, the non-filtered UniProtKB/Swiss-Prot, BLOSUM62, and 2 alignment iterations resulted in an AUC_MoRF_ of 0.422, which is equivalent to 0.578. These three scores were normalized and combined using Bayes rule to generate the *initial conservation propensity score*, *ics* (Fig 2 (C)). Although these three conservation values resulted from the alignment to two different databases, they are still highly related. Nonetheless, the *ics* generated from integrating these three scores provided a higher AUC_MoRF_ than any of its subcomponents, and *ics* performs more consistently over various query sequences.

Next, the *MoRF conservation propensity score*, *mcs*, was determined. Its conception is based on the assumption that binding residues are the most conserved in MoRFs (see Methods: “Binding residues and conservation” for validation). The idea is to have a smooth conservation score such that disordered segments with an average disorder score > *disorder*_*1*_ and more conserved residues than a certain minimum number (i.e. with *ics* > *Conservation*_*1*_) are given high *mcs* scores (scenario 1). In addition, structured segments with an average disorder scores < *disorder*_*2*_ and all their residues’ *ics* < *Conservation*_*2*_ are given low *mcs* scores (scenario 2). The appropriate window sizes and thresholds for these scenarios were learned by testing many possible permutations and selecting those that generate the highest weighted AUC_MoRF_ on TRAINING [16] (Fig 2 (D)). As a result, *mcs* is calculated in the two scenarios as follows: In scenario 1, if a residue r is at the center of a disordered window of 7 residues with an average normalized predicted disorder score > 0.45 and the window includes 3 or more conserved residues with an *ics* > 0.45, then this residue’s *mcs* is assigned to be equal to the average *ics* of the conserved residues. Furthermore, for every extra conserved residue above 3 in that window, a square root function is applied twice to further increase *mcs*. In scenario 2, if a residue r is at the center of a structured window of 15 residues with an average normalized predicted disorder score < 0.45 and no residue within the window has an *ics* > 0.60, then this residue’s *mcs* is assigned to be equal to its *ics* multiplied by the window’s average normalized predicted disorder score. For all other residues that do not fall under scenario 1 or 2, *mcs* is equal to *ics*. (For learning appropriate window sizes and other thresholds, please see: *Learning the MoRF conservation propensity score thresholds values* section in supplement).

### Binding residues and conservation

To validate the assumption that residues involved in binding are more conserved than other residues in MoRFs, we measured the distances between MoRF residues and the surface of the binding partner using coordinates for the MoRF complex structures of the TEST464 dataset collected from the Protein Data Bank [27]. When we separated the MoRF residues into those that are in close proximity to the binding partner and those that are not, we found a significant difference between their median conservation (*ics*) values (distance >5Å *ics* = 0.536 ± 0.005; < = 5Å *ics* = 0.568 ± 0.004; *p* value < 2.2x10^-16^). The confidence intervals for the medians are calculated as $1.58\times IQR/\sqrt{n}$, where IQR is the interquartile range. The IQR is calculated as the difference between the first and third quartile of a distribution of n values as defined in the *boxplot*.*stats* function of R [28] (see “S1 Excel” for data and example calculation). The *p* value is calculated using the Wilcoxon test in R. The distances measured correspond to those between the beta-carbon of the MoRF residues and the closest atom on the surface of the binding partner. The same trend was observable across a range of cut-off values (not shown). The lower conservation scores of the residues above the distance cut-off suggest that residues further away from the interaction surface are less conserved. We also measured the change in relative accessible surface area (rASA) upon binding using NACCESS [29]. The change in rASA of each residue was calculated by subtracting the rASA in the bound state from the rASA in the unbound state, where the unbound structure was obtained by removing all the atoms of the binding partner from the protein complex structure. Once again, we separated the MoRF residues into two groups for comparison. The group of residues that have a change in rASA greater than 20 percent upon binding has a higher median *ics* compared to the remaining residues (> 20% change in rASA *ics* = 0.570 ± 0.004; < = 20% change in rASA *ics* = 0.537 ± 0.005; *p* value < 2.2x10^-16^). This difference in *ics* values suggests that residues that contribute more to the interface surface tend to be more conserved.

### Transforming data to normality (Normalization)

In this work, we utilized a number of component predictors to predict scores for features associated with MoRFs (eg. MoRF_CHiBi_, ESpritz_D, *ics* … etc). Then, we hierarchically integrated these predicted scores into a single propensity score that reflects the likelihood of each residue to be a MoRF residue. Because these component scores are positively correlated with the probability of MoRFs, we chose to integrate them using Bayes rule. Ideally, Bayes rule should be used to combine real probabilities. However, the scores we are integrating predict features that unequally reflect the likelihood of MoRFs. As a result, these component scores do not reflect real MoRF probabilities and include extreme values, which are close to zero or one. These extreme values dominate the outcome of Bayes rule and mask the effect of other features. Using TRAINING, a Map_D_ function was created for each input feature D that transforms its propensity scores from its unknown distribution U_D_ to approximately fit a Gaussian probability density function specified by the normal distribution N(μ = 0.5, σ^2^ = 0.01) while preserving their cumulative values. The cumulative value of the feature D score for a residue x, S^(UD)^_x_, is the probability of a residue in TRAINING to have a propensity score less than or equal to S^(UD)^_x_. Thus, the normalized score S^(ND)^_x_ for S^(UD)^_x_ is:

$$S^{\left(ND\right)}{}_{x}=\;{Map}_{D}(S^{\left(UD\right)}{}_{x})$$

The Map_D_ function for each feature is then used to preprocess the corresponding feature scores of query sequences. (For the implementation procedure, please see: “*Defining MapD function and normalization* section in S1 Text”). For this article, this process of transforming data to a normal distribution is what we call normalization.

### Performance evaluation

We used AUC, the area under the receiver operator characteristics curve (ROC), to evaluate the performance of MoRF_CHiBi_Web_ and compared it with that of MoRF_CHiBi_, MoRFpred and ANCHOR. Even though AUC is a common evaluation metric for prediction performance, it only provides an overall assessment about the separation of the two classes, which are MoRFs and non-MoRFs in this case. However, in most scenarios, we are only interested in high confidence predictions at some high threshold values represented by the lower left corner of the ROC curve. Thus, we also compared false positive rates, FPRs, at different true positive rate values, TPRs, for each predictor. TPR is defined as TPR = TP / N_MoRF_ where true positive, TP, is the number of accurately predicted MoRF residues, and N_MoRF_ is the total number of experimentally verified MoRF residues. False positive rate is defined as FPR = FP / N_non-MoRF_ where FP is the number of inaccurately predicted MoRF residues, and N_non-MoRF_ is the total number of non-MoRF residues. On occasion, to maintain consistency with evaluations presented by other groups, we also used sensitivity and specificity where sensitivity is equal to TPR and specificity is 1 –FPR.

## Results

When comparing the performance of different machine learning predictors, it is crucial for the test data, or homologs of test data sequences, not to overlap with data used in the training of the predictors. In our case, MoRF_CHiBi_Web_ was trained on the same TRAINING set used by MoRFpred and MoRF_CHiBi_, so we can make detailed performance comparisons with these predictors using the two datasets, TEST464 and EXP53, described in the Datasets section in Methods. Both TEST464 and EXP53 consist of sequences with less than 30% identity to those in TRAINING. These two test sets are also used for comparison with ANCHOR since it has minimal reliance on its training set. Note that all the predictors above are trained on short MoRFs with up to 30 residues, but EXP53 also includes MoRFs that are longer than 30 residues. Thus, when testing the predictors’ performance on EXP53, we also provided a separate performance evaluation for short and long MoRFs. Table 1 shows the AUC values for each of the four predictors and reveals that MoRF_CHiBi_Web_ outperforms the other ones.

**Table 1:** AUC Results.

Data	MoRF_CHiBi_Web_	MoRF_CHiBi_	MoRFpred	ANCHOR
TEST464	0.806	0.748	0.675	0.605
EXP53_All	0.789	0.714	0.620	0.615
EXP53_Short	0.877	0.790	0.673	0.683
EXP53_Long	0.751	0.681	0.598	0.586

AUC values of the four MoRF predictors using the TEST464 and EXP53 data sets. For EXP53_Short, only MoRF sections with up to 30 residues are considered as MoRFs, while longer MoRF sections were masked out. Similarly, for EXP53_Long, only MoRF sections longer than 30 residues are considered, while shorter MoRFs were masked out.

**Fig 3:**
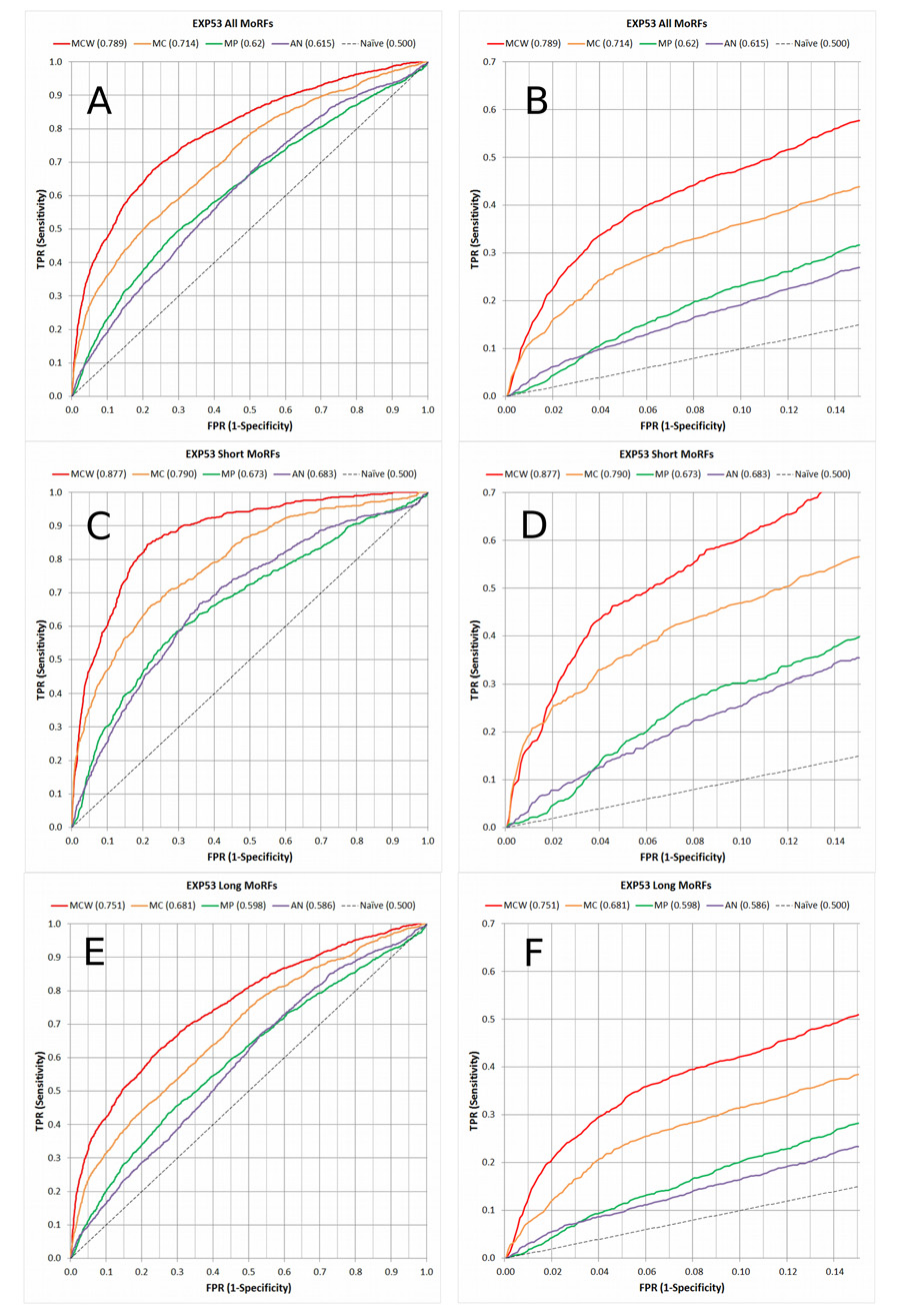
Full ROC curves (A, C and E) and their lower left corners (B, D and F) for the EXP53 dataset. ROC curves in A and B show the MoRF prediction performance for all MoRFs. C and D show the prediction performance of short MoRFs with up to 30 residues while longer MoRFs are masked out, and E and F show the performance of long MoRFs with more than 30 residues while shorter MoRFs are masked out. Vertical axis is the true positive rate (Sensitivity) and horizontal axis is the false positive rate (1-Specificity). MoRF_CHiBi_Web_ (MCW) is in red, MoRF_CHiBi_ (MC) in orange, MoRFpred (MP) in green, and ANCHOR (AN) in purple. The dashed line (Naïve) represents a random classifier. AUC values are in parentheses next to each label.

ROC curves generated for TEST464 (Fig C in S1 Text) and EXP53 (Fig 3) show that MoRF_CHiBi_Web_ has a higher specificity (1- FPR) at any level of sensitivity (TPR) when compared to ANCHOR and MoRFpred. All four predictors performed better on EXP53_Short than on TEST464. This difference is expected because each sequence in TEST464 is labeled with only one MoRF, despite the fact that some sequences contain more MoRFs. For example, all MoRFs in the protein p53 are annotated on a single sequence in EXP53, whereas TEST464 contains eight almost identical sequences of p53 with only one annotated MoRF in each, which were all identified based on different PDB structures (Uniprot: P04637, Q2XSC7, Q2XN98, Q9TTA1 … etc.). Therefore, evaluations with the more sparsely annotated sequences of TEST464 results in some true MoRF residues with high prediction scores being erroneously treated as non-MoRF resides. Hence, we think that the overall performances are more accurately represented by test results on EXP53 than on TEST464.

When compared to MoRFpred and ANCHOR, MoRF_CHiBi_Web_ produces less than half the false positive rate for the same true positive rate at practical threshold values (Table 2).

**Table 2:** FPR as a Function of TPR.

TPR (Sensitivity)	FPR (1—Specificity)			
	MoRF_CHiBi_Web_	MoRF_CHiBi_	MoRFpred	ANCHOR
0.2	0.016, 0.015, 0.018	0.030, 0.010, 0.038	0.081, 0.059, 0.099	0.103, 0.070, 0.128
0.3	0.032, 0.022, 0.041	0.064, 0.035, 0.091	0.140, 0.095, 0.168	0.173, 0.118, 0.217
0.4	0.060, 0.034, 0.083	0.124, 0.065, 0.165	0.218, 0.154, 0.252	0.263, 0.175, 0.310
0.5	0.113, 0.061, 0.144	0.201, 0.117, 0.264	0.306, 0.227, 0.353	0.345, 0.249, 0.398

*FPR as a function of TPR computed on the EXP53 set (All MoRFs*, *short MoRFs*, *long MoRFs) for MoRF*_*CHiBi_Web*_, *compared to MoRF*_*CHiBi*_, *MoRFpred*, *and ANCHOR at different TPR cut off values*.

**Fig 4:**
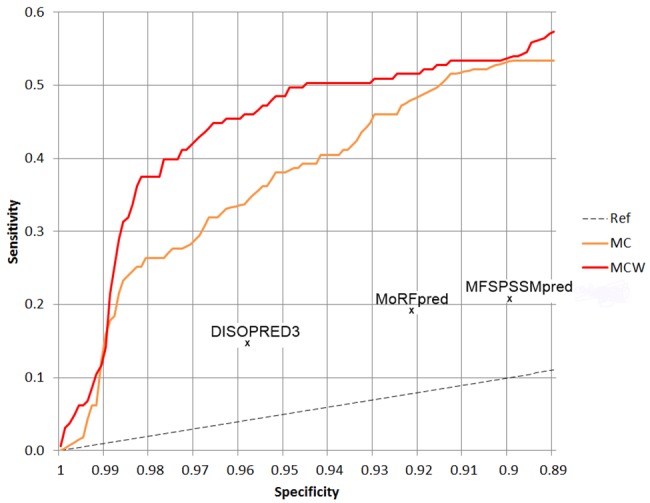
Comparing the performance of MoRF_CHiBi_Web_ (MCW in red), MoRF_CHiBi_ (MC in orange), DISOPRED3, MFSPSSMpred and MoRFpred using EXP9. Curves of sensitivity as a function of specificity for MoRF_CHiBi_Web_ and MoRF_CHiBi_ are computed on a set of 9 protein sequences collected by Jones and Cozzetto [15]. The data points for each of the three predictors, DISOPRED3, MFSPSSMpred and MoRFpred, are from published results of Jones and Cozzetto [15].

As MFSPSSM and DISOPRED3 were trained on a large training set that includes all the sequences of TEST464 and many of the sequences in EXP53 (or homologs thereof), we compared the performance of MoRF_CHiBi_Web_ with the results of DISOPRED3, MFSPSSMpred and MoRFpred that were reported by Jones and Cozzetto [15] based on the EXP9 set. This set contains experimentally validated MoRFs that have no sequence homology with MoRFs used in the training of all these predictors and thus MoRF_CHiBi_Web_. For each predictor, only one data point (i.e. specificity, sensitivity) was provided by Jones and Cozzetto [15]. On this small test set, MoRF_CHiBi_Web_ also achieves higher sensitivity (TPR) for a given specificity (1-FPR) when compared to the other predictors (Fig 4).

As MoRF predictors are often used to screen large sets of proteins, we were also interested in their efficiencies. Prediction times of MoRF_CHiBi_ and ANCHOR, both of which do not require PSI-BLAST alignments, were tested by scoring the entire TEST464 set on an Intel core i7, 3.44G desktop. MoRF_CHiBi_Web_, MoRFpred, DISOPRED3 and MFSPSSMpred were tested by repeatedly submitting a single sequence from the TEST464 set (Uniprot:Q38087) with 903 residues to their web sites and then selecting the fastest outcome. ANCHOR is the fastest with a processing speed of 4*10^6^ r/m (residues/minute), MoRF_CHiBi_ came in second with 6*10^3^ r/m, and MoRF_CHiBi_Web_ is third with 536 r/m. MFSPSSMpred processed 135 r/m, MoRFpred 41 r/m and DISOPRED3 13 r/m. This comparison might not be entirely fair as the underlying hardware of web-based predictors is unknown. Nevertheless, the comparison of computational costs of the tools that depend on PSI-BLAST alignment clearly shows that MFSPSSMpred, MoRFpred and DISOPRED3 are significantly slower than the predictor introduced here.

## Discussion

We presented a new web-based approach, MoRF_CHiBi_Web_, for predicting MoRFs within protein sequences. We compared its performance to that of MoRFpred, MoRF_CHiBi_ and ANCHOR using two different test sets: TEST464 with 464 sequences and EXP53 with 53 sequences that contain only experimentally validated MoRFs. The results demonstrate that MoRF_CHiBi_Web_ outperforms all three predictors. Furthermore, MoRF_CHiBi_Web_ generates less than half the false positive rate of other predictors at most cut-off values. Because MFSPSSMpred and DISOPRED3 use a different set of training data, we have to rely on a small set of only 9 sequences, EXP9, collected by the authors of the latter to compare the performance of these two predictors with MoRF_CHiBi_Web_. Although results from such a small dataset might not accurately reflect the actual performance of these predictors, MoRF_CHiBi_Web_‘s significantly higher sensitivity at selected specificities shows a consistent advantage over these predictors.

The high accuracy of MoRF_CHiBi_Web_ can be attributed to the use of three mostly independent sources of information: sequence similarity and contrast in amino acid composition, disorder content of the MoRF-harbouring segment, and conservation of the MoRF sequence. The first source has been exploited in a stand-alone predictor (MoRF_CHiBi_) that we developed previously [16]. As MoRF_CHiBi_ only considers local sequence information, it does not utilize information about the level of disorder of the protein segments in which the putative MoRFs are located. The location of MoRFs within IDRs is part of their definition and distinguishes them from a similar and overlapping class of protein sequences known as short linear motifs (SLiMs). SLiMs are sequences 3 to 10 residues in length that have been found to be enriched in IDRs and bind specific protein domains [30]. However, SLiMs are also found in structured domains, as exemplified by PY motifs buried in structured proteins that get exposed and recognized by E3 ubiquitin ligase upon heat shock [31]. MoRF_CHiBi_ was trained and tested on MoRFs only and, therefore, the inclusion of disorder information was necessary and rewarding. Furthermore, residues in IDRs are known to be less conserved on average than those in MoRFs [18, 19]. Therefore, MoRF_CHiBi_Web_ was also designed to exploit the difference in conservation to improve predictions.

**Fig 5:**
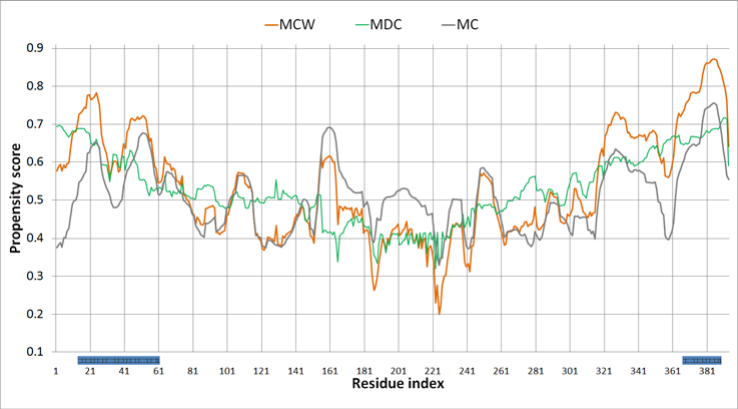
MoRF_CHiBi_Web_ Propensity scores for the protein p53. Propensity scores for the protein p53 by MoRF_CHiBi_Web_ (MCW) in orange, MoRF_CHiBi_ (MC) in gray and MoRF_DC_ (MDC) in green. The 2 verified MoRF sections in our EXP53 test set are marked in blue below the chart.

The improvement derived from the addition of information on both intrinsic disorder and conservation to MoRF_CHiBi_ can be exemplified in the case of the protein p53 (Fig 5). This protein contains two main regions where the existence of MoRFs has been verified: one in the N-terminal region and the other in the C-terminal region. The propensity scores generated by MoRF_CHiBi_ correctly identified the N and C-terminal MoRFs but they also incorrectly identified the region around residue 161 as a MoRF segment, as shown by the grey curve in Fig 5. The region between residues 95 and 292 is known to be a globular domain (PDB: 1gzh; [32]). MoRF_DC_ keeps the propensity scores low in this globular region, which suppresses the MoRF_CHiBi_Web_ scores at the region around position 161 relative to the scores of the N and C-terminal MoRF regions. The addition of disorder and conservation information is thereby able to broaden the gap in propensity scores between MoRF and non-MoRF regions, which allows for a more stringent cutoff that generates a lower false positive rate. Interestingly, the remaining false positive region near the C-terminus is also known to make interactions, but it has not been verified to be intrinsically disordered [17].

As some predictors have been trained on all available MoRFs, it is important to discuss issues regarding scoring bias towards the training data. In general, MoRFs in query sequences that are identical or very similar to those in the training set are scored favorably compared to other MoRFs. This scoring bias can often be misleading as we naturally expect the score quality to be consistent throughout the entire query sequence. Hence, the scoring bias can hamper the identification of novel MoRFs because their scores are overshadowed by those over-scored training MoRFs. Different machine learning tools are affected differently by training data. Support vector machines with RBF kernels are known to be prone to over-scoring their training data [33], whereas over-scoring is minimal with the use of linear and sigmoid kernels. One of MoRF_CHiBi_Web_‘s components is a SVM-RBF model, namely the SVM_T_ model in MoRF_CHiBi_. However, the potential for bias brought on by SVM_T_ is mitigated by contributions from the other models, MoRF_CHiBi_‘s SVM_S_ and MoRF_DC_’s conservation and disorder components, which are all less susceptible to over-scoring. Specifically, MoRF_CHiBi_‘s SVM_S_ employs a sigmoid kernel and was trained on synthetic data. Furthermore, MoRF_DC_’s conservation component has very limited reliance on training and its disorder component comes from ESpritz, which is based on a neural-network model that was trained on an independent dataset from DisProt. Using Bayes rule to integrate multiple propensity scores generated by different predictors targeting different features using different machine learning tools provides MoRF_CHiBi_Web_ with less potential for biased scoring compared to predictors that are based on a single machine learning tool.

In summary, we introduced a new MoRF predictor, MoRF_CHiBi_Web_, which is based on hierarchically incorporating several independently computed propensity scores of features associated with MoRFs. MoRF_CHiBi_Web_ is fast and its predictions include less than half the false positives compared to all available MoRF predictors. Regarding its usage, we like to stress that categorical prediction by assigning a static cut-off value can be a misleading oversimplification. Yet, if users need such a cut-off, we suggest a value of 0.66. At this cut-off, MoRF_CHiBi_Web_ achieved sensitivities of 0.482, 0.615, 0.425 for all_MoRFs, short_MoRFs, and long_MoRFs from EXP53, respectively, at a specificity of 0.897.

## Supporting Information

S1 Excel Binding residues and conservation. Excel file for data and example calculation.
(XLSX)

S1 Text Supporting Information: Computational Identification of MoRFs in Protein Sequences Using Hierarchical Application of Bayes Rule. (DOCX)

S2 Text EXP53 annotated test data. (TXT)
