# Supplementary material for: Computational Identification of MoRFs in Protein Sequences Using Hierarchical Application of Bayes Rule
Source: PLoS One. 2015 Oct 30;10(10):e0141603. doi: 10.1371/journal.pone.0141603 (PMC4627796; doi:10.1371/journal.pone.0141603)
Supplement: S1 Text — (DOCX) [file pone.0141603.s002.docx]

**Supporting Information: Computational Identification of MoRFs in Protein Sequences Using Hierarchical Application of Bayes Rule**

Nawar Malhis^1*^, Eric T. C. Wong^1,2^, Roy Nassar^1^, and Jörg Gsponer^1,2*^

^1^Centre for High-Throughput Biology, University of British Columbia, Vancouver, BC, Canada.

^2^Department of Biochemistry and Molecular Biology, University of British Columbia, Vancouver, BC, Canada.

* Corresponding authors

[nmalhis@chibi.ubc.ca](mailto:nmalhis@chibi.ubc.ca) (NM)

[gsponer@chibi.ubc.ca](mailto:gsponer@chibi.ubc.ca) (JG)

**Defining Map_D_ function and normalization**

The idea is to construct a function Map_D_ that maps a set of feature scores, D, from its unknown distribution U_D_ to a Gaussian distribution N(µ, σ^2^), while preserving the cumulative values of this feature’s scores (see fig A). In other words, we want to transform the distribution of the set of scores while keeping their rank order the same. Thus, if *x ϵ D*, *y ϵ D*, and *x > y*, then *Map_D_(x) > Map_D_(y)*.

**
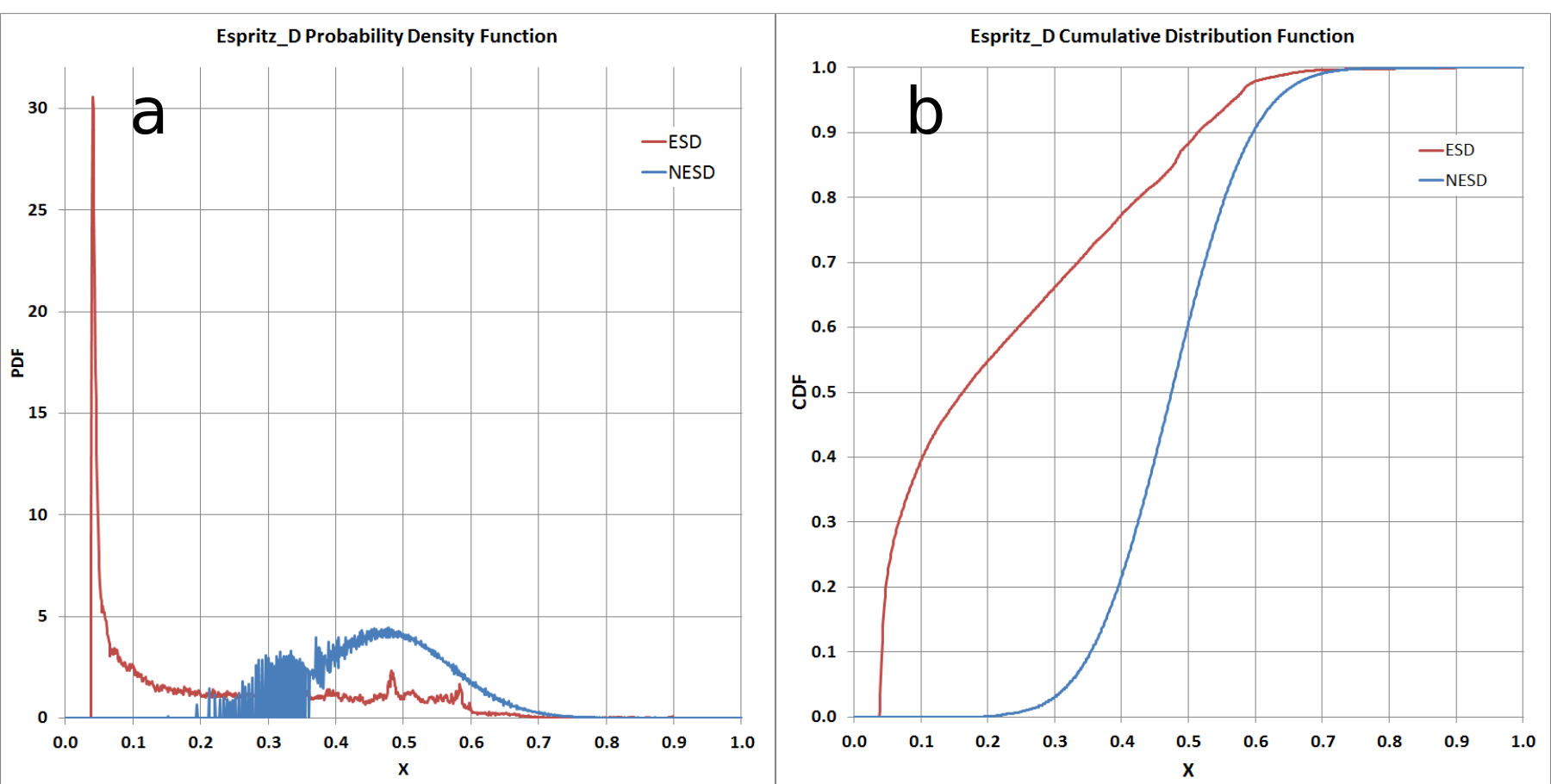
**

**Fig A. The ESpritz_D scores before and after normalization.**

The ESpritz_D scores for the NRT (the non-redundant subset of TRAINING) before normalization, ESD, in brown, and after normalization, NESD, in blue. (A) The probability density function. (B) The cumulative distributions function. The fluctuation at the left part of the probability density function of NESD is a result of scaling the scores with equal values at the low end of the ESD distribution.

The expected value µ is set to be the Bayes rule identity element, 0.5, so that rescaled feature scores with the value 0.5 will have no effect on the outcome of Bayes rule. The standard deviation σ should be set to reflect that particular feature’s ability to predict MoRF residues, such that features with higher MoRF predictive power are normalized using higher standard deviation values to increase their contribution to the outcome of Bayes rule. AUC_MoRF_ is used to assess a feature’s ability to predict MoRF residues. Thus, when Bayes rule is used to incorporate two sets of scores S_1_ and S_2_, if AUC_MoRF_ for S_1_ is higher than that of S_2_, then the σ_1_ used for normalizing S_1_ should be higher than that used for S_2_. Consequently, standard deviation values need to be learned from TRAINING. However, it happened by chance that every time we applied Bayes rule in our procedure, both of our input sets have approximately equal AUC_MoRF_ values (using TRAINING). Therefore, we used the same standard deviation value (0.1) for all input sets of Bayes rule. Note that while the relative σ values between the input sets of Bayes rule affect the relative contribution by each of these sets, the only constraint on the absolute value of σ is to have the normalized input scores spread appropriately within the range zero to one, since we normalized the outcome after each application of Bayes rule.

**Implementation:** First, Map_D_ functions are learned from a training data, and then they are used to pre-process features scores for query sequences. For training, we used the set of TRAINING non-redundant sequences, NRT, which includes 283 sequences that have at most 90% identity to each other, accounting for a total of ~187,000 residues.

Note that an optimal mapping to Gaussian distribution with a complete preservation of the cumulative values can only be assured when there are no equal scores in D. This is not always true with our data as the low precision of some scores results in significant score overlaps. For example, the PSSM *weighted observed percentage of the query sequence residue rounded down* scores is an integer in the range [0 – 100], thus, overlapping scores will inevitably exist when D has more than 101 values, which leads to a suboptimal mapping.

Constructing the Normalization *Map_D_* function for a set of scores D:

1. Scores in D are rescaled to the range 0.05 to 0.95:

- We identified the highest (*Max*) and lowest (*Min*) scores in D.
- For each score in D, we subtracted *Min*, divided by (*Max* – *Min*), multiplied by 0.9, and added 0.05.

1. An array, *Scores,* holds the scores of D, sorted from smallest to largest.
2. An array, *GaussianCDF*, of size 10,000 is constructed to hold the values of the cumulative distribution function, CDF, for N(0.5, 0.01), such that the value at any location (i) in *GaussianCDF*, is equal to the value of CDF(i / 10,000).
3. A second array, *map*, of size 10,000 is also constructed such that for every location i in *GaussianCDF*,

- *map*[*Scores*[ *GaussianCDF*[i] * |D| ] * 10,000 ] = i /10,000.
- *Map[0] is set to 0.01, Map[500] is set to 0.05, Map[9500] is set to 0.95 and map[10,000] is set to 0.99.*

1. The values of those locations in the *map* array that are not assigned in step 4 are computed linearly based on the values around them.

*Min*, *Max* and *map* for each feature are saved and then used to normalize that feature scores in query sequences. Normalization of the query sequences’ features scores is done in two steps: First, scores are rescaled using *Min* and *Max* associated with that feature as explained above in “Constructing the Normalization MapD function” step 1. Then, the normalized value for each rescaled score (x) can be obtained from the map cell at index x * 10,000.

Please see fig B for examples of the resulting map arrays. Note that the MoRF_CHiBi__*_Web_* line is approximately linear, which indicates that its input is almost identical to its output. This is expected as the final step in MoRF_CHiBi_Web_ generation is joining two largely independent sets that have previously been normalized.

**
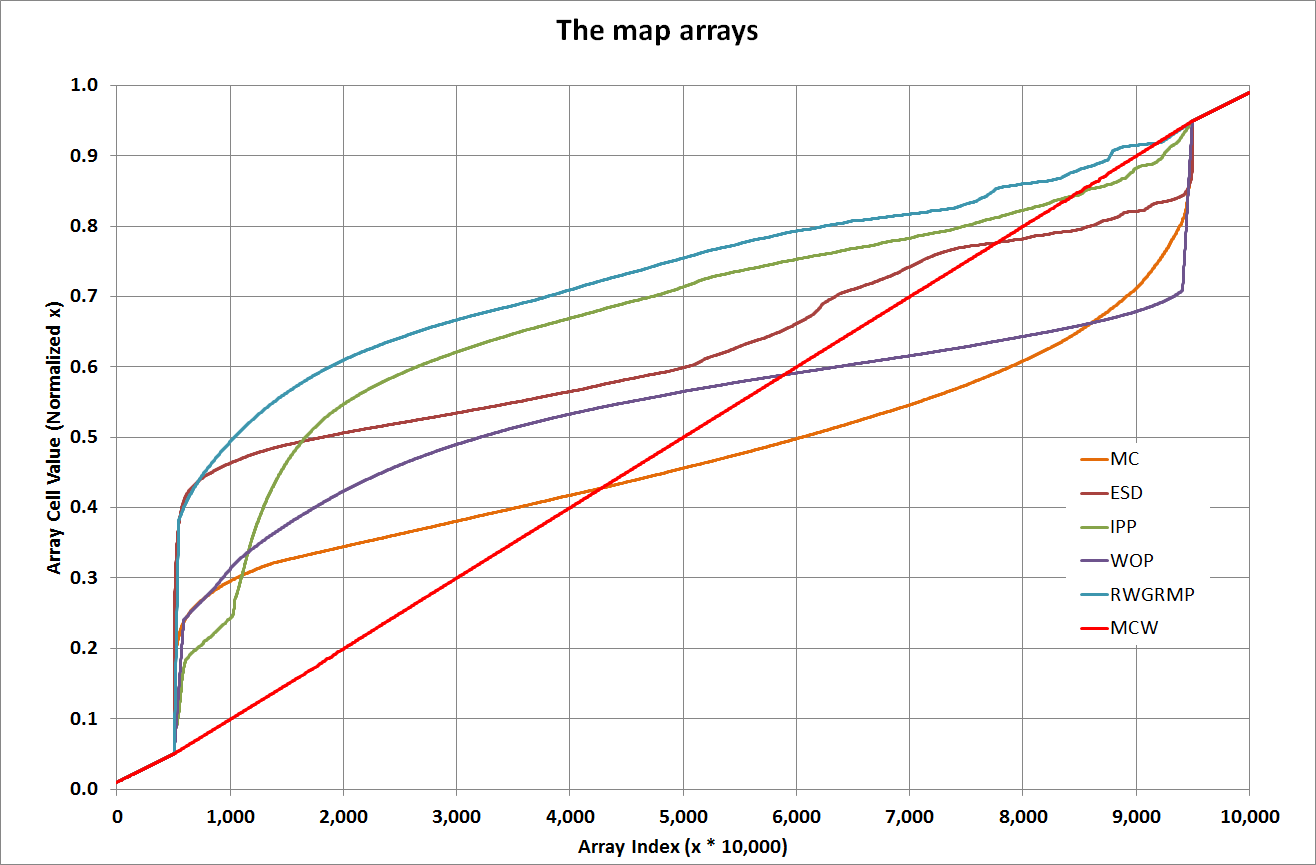
**

**Fig B. Some examples of the calculated map arrays.**

The horizontal axis is the array index and the vertical axis is the value of the array cell at that index. MC for MoRF_CHiBi_, ESD for ESpritz_D, MCW for MoRF_CHiBi_Web_, IPP for PSSM information per position, WOP for weighted observed percentage of the query sequence residue rounded down, and RWGRMP for the relative weight of gapless real matches to pseudocounts.

**Learning the MoRF conservation propensity score thresholds values**

The appropriate window sizes and other thresholds were learned from TRAINING by testing all possible permutations for the seven variables under two scenarios and selecting those that generate the highest weighted AUC_MoRF_ [1] for MoRF_CHiBi_Web_. Annotated MoRF residues are used as positive class, and the remaining residues are used as negative class. The two scenarios are:

1. Disordered segments with an average disorder score > *disorder_1_* and a minimum number of conserved residues equal to (or greater than) *mncr,* that have *ics* > *conservation_1_* are given high *mcs* scores. For the four parameters in this scenario, the values tested are:
   1. The *window_1_* sizes ϵ {3, 5, 7, 9},
   2. The *disorder_1_* values ϵ {0.35, 0.40, 0.45, 0.50, 0.55},
   3. The *conservation_1_* values ϵ {0.35, 0.40, 0.45, 0.50, 0.55}, and
   4. The *mncr* ϵ {1, 2, .. ((*window_1_* *size* - 1) / 2 + 1)}.
2. Structured segments of length *window_2_* size with average disorder scores < *disorder_2_* and all their residues’ *ics* < *conservation_2_* are given low *mcs* scores. The values used for learning this scenario’s three parameters are:
   1. The *window_2_* sizes ϵ {7, 9, 11, 13, 15, 17},
   2. The *disorder_2_* values ϵ {0.40, 0.45, 0.50, 0.55, 0.60} and
   3. The *conservation_2_* values ϵ {0.40, 0.45, 0.50, 0.55, 0.60, 0.65}.

**
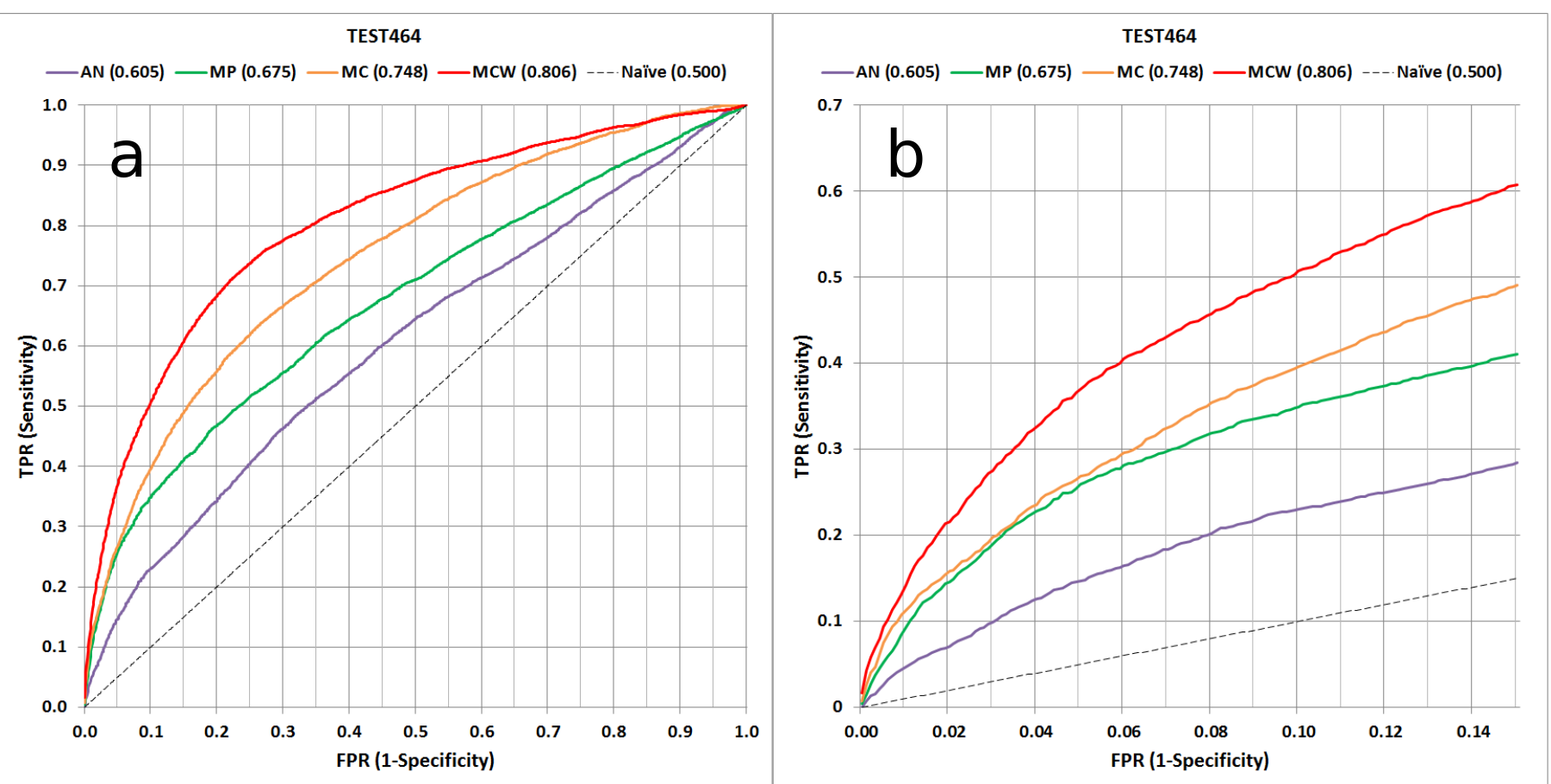
**

**Fig C. ROC Curves for the TEST464 dataset.**

Full ROC curves (a) and the lower left corners (b). Vertical axis is the true positive rate (Sensitivity) and horizontal axis is the false positive rate (1-Specificity). MoRF_CHiBi_Web_ (MCW) is in red, MoRF_CHiBi_ (MC) in orange, MoRFpred (MP) in green, and ANCHOR (AN) in purple. The dashed line (Naïve) represents a random classifier. AUC values are in parentheses next to each label.

**Dataset for comparison between conservation scores, distances, and relative accessible surface area**

We downloaded the PDB files corresponding to the TEST464 dataset. We skipped PDB structures where the target protein chain contained non-canonical or unknown residues, since they may cause errors in accessible surface area calculations. We also skipped structures where the number of residues in the coordinate section of the PDB file does not directly match the number of residues defined as part of the MoRF in our dataset. The resulting dataset is provided in an Excel file. The spreadsheet named “Complete with PDBID” contains the full dataset. The spreadsheet named “Modified for R” contains a slightly smaller dataset created as a result of removing some residues with an incomplete set of data points, which could be caused by missing coordinates in the PDB file that hampered calculations.

# References

1. Malhis N and Gsponer J. Computational Identification of MoRFs in Protein Sequences. *Bioinformatics* 2015 Jan 30; 31 (11): 1738-1744*.*
